# Fission of Lipid-Vesicles by Membrane Phase Transitions in Thermal Convection

**DOI:** 10.1038/s41598-019-55110-0

**Published:** 2019-12-11

**Authors:** Patrick W. Kudella, Katharina Preißinger, Matthias Morasch, Christina F. Dirscherl, Dieter Braun, Achim Wixforth, Christoph Westerhausen

**Affiliations:** 1 Chair for Experimental Physics I, University of Augsburg, Augsburg, Germany; 2 Center for NanoScience (CeNS), Ludwig-Maximilian University Munich, Munich, Germany; 3 Systems Biophysics, Ludwig-Maximilian University Munich, Munich, Germany; 4 Chair for Physiology, University of Augsburg, Augsburg, 86159 Germany

**Keywords:** Membrane biophysics, Biomaterials - cells, Biological physics

## Abstract

Unilamellar lipid vesicles can serve as model for protocells. We present a vesicle fission mechanism in a thermal gradient under flow in a convection chamber, where vesicles cycle cold and hot regions periodically. Crucial to obtain fission of the vesicles in this scenario is a temperature-induced membrane phase transition that vesicles experience multiple times. We model the temperature gradient of the chamber with a capillary to study single vesicles on their way through the temperature gradient in an external field of shear forces. Starting in the gel-like phase the spherical vesicles are heated above their main melting temperature resulting in a dumbbell-deformation. Further downstream a temperature drop below the transition temperature induces splitting of the vesicles without further physical or chemical intervention. This mechanism also holds for less cooperative systems, as shown here for a lipid alloy with a broad transition temperature width of 8 K. We find a critical tether length that can be understood from the transition width and the locally applied temperature gradient. This combination of a temperature-induced membrane phase transition and realistic flow scenarios as given *e.g*. in a white smoker enable a fission mechanism that can contribute to the understanding of more advanced protocell cycles.

## Introduction

Before life on earth emerged, only small molecules existed. The development of complex structures or already simple reactions were unlikely and energetically unfavorable simply due to the assumed very dilute settings of early earth^1,2^. Physical non-equilibrium settings can have a profound influence here: sub-sea hydrothermal microenvironments^3^ like pores in submerged volcanic rock provide temperature cycling systems and allow for strong accumulation, as the cooperation of convective flow and thermophoresis can enlarge molecule- and particle-concentrations by several orders of magnitude^4^.

An important concept in early earth’s cell evolution is compartmentalization. Following Blain, Szostak^5^ and Nourian, Danelon^6^ a compartment such as a lipid membrane provides a selective barrier between the inner proto-cell content and the surrounding environment. Schrum *et al*. describe a pathway for lipid membrane vesicles made from fatty acids known as prebiotically plausible membrane building blocks^7^ to more sophisticated phospholipids. They then raise the question how such structurally simple protocells could accomplish essential membrane functions know from modern cells.

In a model cell cycle for vesicles the two phases, *formation* and *inclusion* of diluted substances, are well understood^5^. The *division*-phase has been the subject of several studies^5,6,8,9^. This division is necessary to redistribute enclosed material or reaction products formed in the protocell. While modern cell division is a complicated mechanism mediated by many complex proteins, an early earth protocell must have had a simple fission mechanism. A basic cell division-like fission could have been driven by the environment and membrane properties only, without any active contribution by the protocell itself. The temperature-dependent hydrophobic effect drives the self-assembly of vesicles from dissolved lipids above a critical concentration and e.g. for dipalmitoylphosphatidylcholine (DPPC) based membranes also causes complex reshaping of the vesicle membrane during several phase transitions^10,11^. Temperature induced morphological transitions of DPPC vesicles have been shown by Leirer *et al*. in absence of additional shear flow. The vesicles showed a pear-shaped morphological transition of previously spherical DPPC vesicles by the DPPC L_β’_ - L_α_ membrane phase transition^12^ by quick temperature changes in a static setup. Rapid cooling can fission those vesicles, but the resulting new vesicles differ strongly in size compared to the mother vesicle. They do not result in two or more similarly sized vesicles resembling modern cell division. Moreover, such a temperature setup does not resemble scenarios possible on early earth, as closed fluid compartments with rapid temperature change over some seconds without flow do barely exist in nature.

Zhu, Budin, and Szostak show the growth and division of proto-cell membranes under mild flow^9,13^. Vesicle growth is driven by uptake of micelles by small unilamellar vesicles, the resulting aggregates are tube-shaped due to the large surface-to-volume ratio of the integrated micelles. The low permeability of the fatty acid bilayer inhibits the influx of water and therefore their reshaping to spheres. Already a mild fluid flow induces shear forces by which these aggregates are ruptured. The tube-like structures shown by Zhu *et al*. are fragile and too easily divided to be suitable for the robust transport and enclosure of molecules such as DNA. Alternatively, the surface-to-volume imbalance can be achieved by lipid production in the vesicle^14^. Deshpande *et. al* use the osmotic pressure to increase the surface-to-volume ratio and then use an obstacle in a microfluidic channel to mechanically divide DOPC-vesicles. They note, that the success of vesicle-splitting mainly depends on the surface-to-volume ratio of the mother vesicle and a too small ratio leads to complete rupture of the vesicle^15^.

The concept of a primitive cell cycle with division of cell-sized lipid vesicles is modelled in several studies^16^: in all cases the concept introduces an imbalance of vesicle surface and volume^15,17–19^, as shown above, or shear forces and thermodynamic instability as shown by Szostak *et al*. In combination with a spontaneous growth mechanism, this “[…] could lead to a primitive cell cycle controlled entirely by the biophysical properties of the membrane and environmental forces”^17,20^.

When modern cells like *Bacillus subtilis* are treated by antibiotics they lose their cell wall. In turn, their division mechanism (Z-ring) does not work anymore. In order to proliferate, these cells now grow and undergo a shape transformation to pearl or dumbbell-like shapes and split into multiple cells^21^. This observation shows the ability for an alternative self-driven division mechanism in nowadays cells that is comparable to the division of lipid vesicles^22^.

Here, we describe a simple process of splitting Giant Unilamellar lipid Vesicles (GUV) in homogeneously sized daughter vesicles. In our system this process is driven by environmental conditions of flow induced shear forces in a spatial temperature gradient. The vesicles have a stable membrane with low permeability before and after the fission process and hardly lose any membrane area during fission. In contrast to other mechanisms, here no additional chemical or biological effect or mechanism is necessary.

## Results and Discussion

**Figure 1:**
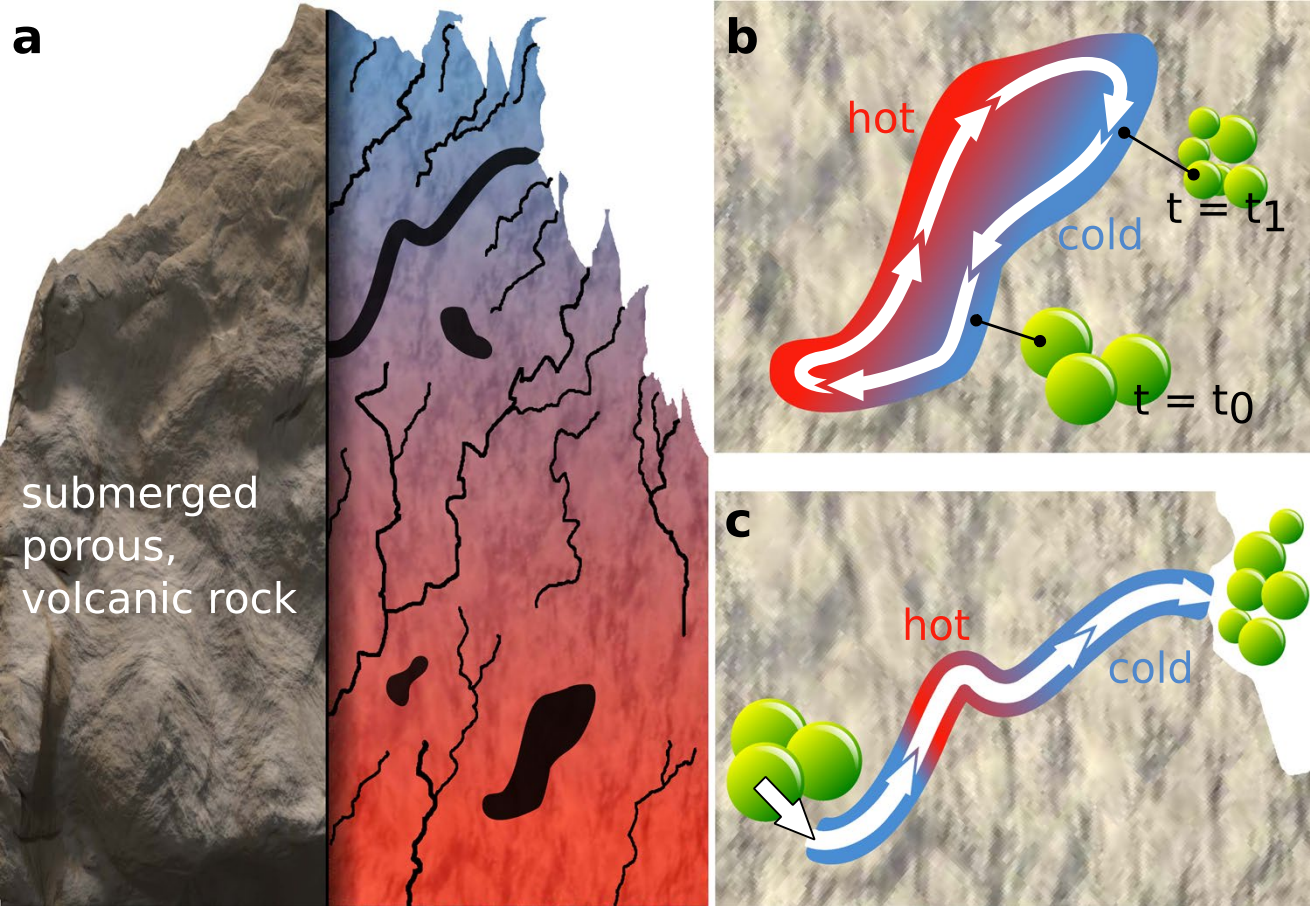
Schematic sketch of a subsea hydrothermal microenvironment, (**a**) Submerged porous (volcanic) rock is a scenario for stable temperature gradients and micro fluidics: water flows through narrow cracks and compartments in the rock where hot parts of thermal vents and the cold ocean create steep temperature gradients on small length scales. (**b**) Vesicle division in a convection chamber that provides temperature driven flow and temperature cycling. (**c**) Vesicle division by a fluid flow profile in combination with temperature induced lipid membrane phase transitions.

In temperature-driven convection chambers we first discovered distinctly deformed DPPC vesicles. The chambers are built to mimic hydrothermal microenvironments^3^ and sustain flow through the applied temperature gradient. In nature comparable chambers are located in rocks that are heated from the inside (by lava or hot gases) and cooled from the outside (ocean). Figure 1 illustrates such a rock as well as possible capillary or chamber geometries (closed and open channels). Keil *et al*. show, that the geometry of a chamber can vary without altering the convection or accumulation effect significantly^4^.

**Figure 2:**
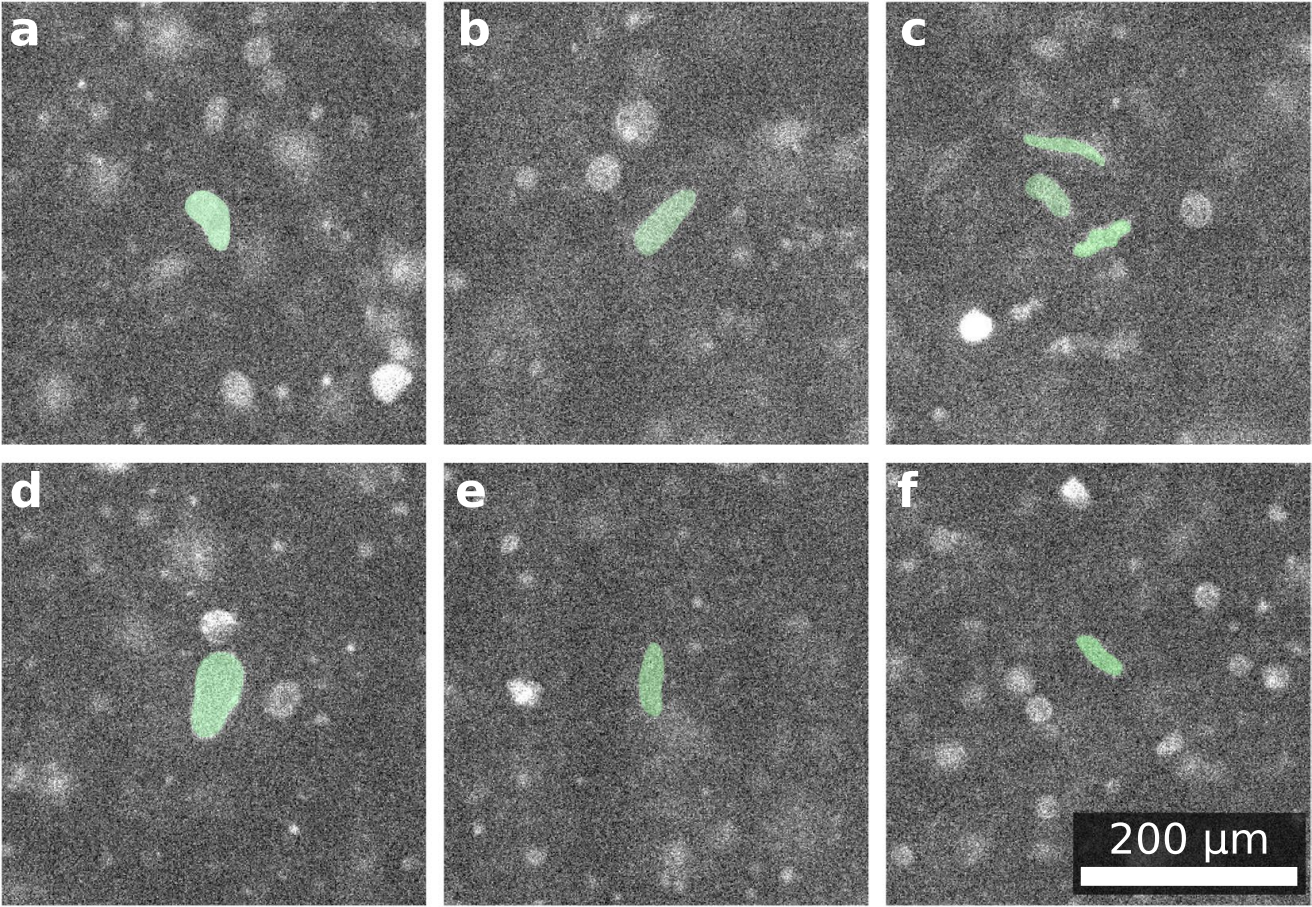
Deformed DPPC-DPPG GUV in a convection chamber: (**a**–**f**) deformed vesicles (guide to the eye: green translucent overlay) in a thermally driven convection chamber. Some vesicles are too small or in regions with less shear force and do not deform. Vesicles do only reshape at temperatures above the membrane phase transition temperature. Due to the shallow thickness of the chamber of 400 µm, vesicles in cold and in hot regions are seen sharp simultaneously with the optics used.

**Figure 3:**
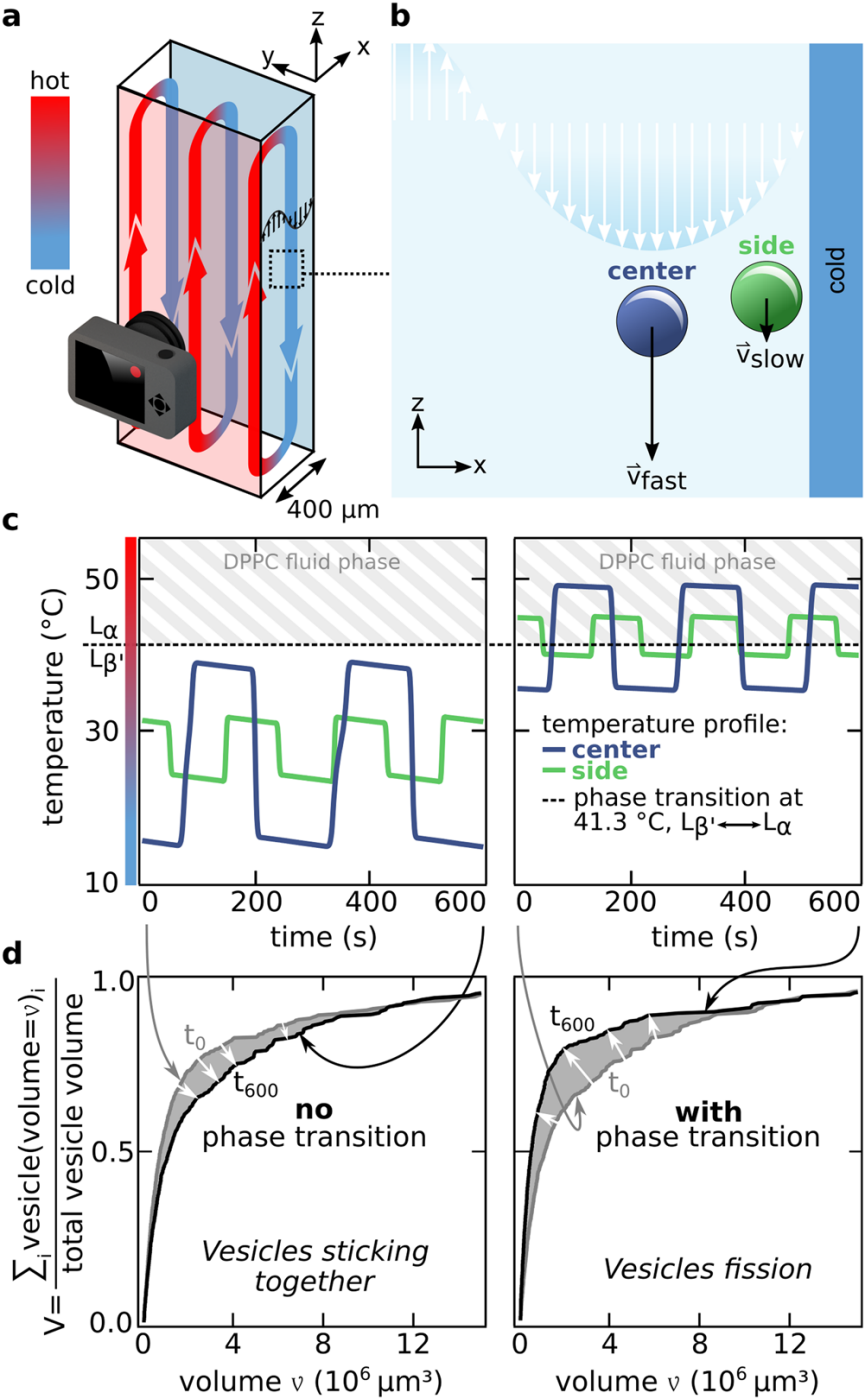
Thermally driven convection chamber: (**a**) The temperature gradient across the 400 µm thick capillary induces a convective flow. Lipid vesicles are carried in this flow and experience hot and cold conditions, as well as shear forces originating from the parabolic flow profile. At the top and the bottom of the chamber, a phase transition of the vesicle membrane can occur. (**b**) Vesicles in the center of the flow profile are moving the fastest. Near the chamber wall fluid flow is slow. This leads to a different number of experienced phase transitions for each vesicle. (**c**) The COMSOL simulation of the flow profile in the chamber overlaid by a particle tracker shows the temperature profile in a stream line over time. For elevated temperatures, the streamline crosses the phase transition temperature of DPPC membranes. To achieve similar mean flow velocities, ΔT of the temperature gradient is reduced to compensate for the lower viscosity of water at higher temperatures. While at lower temperatures the vesicles simply shuttle in the convective flow, at higher temperatures the vesicles experience two phase transitions for each cycle (simulation and particle tracker from Keil^4^). (**d**) The vesicle size distribution in the convection chamber before and after cycling. When the applied temperature range does not include the phase transition temperature (silicon: 13 °C, sapphire: 40 °C, see SI) a small shift in the size distribution towards slightly larger vesicles is visible. This can be understood by vesicles sticking together. For higher temperatures (silicon: 34 °C, sapphire: 50 °C) the size distribution is significantly shifted to smaller sizes.

Figure 2 shows deformed vesicles in the convective flow. The initially spherically shaped vesicles now show non-spherical forms. Some time after the gradient is applied, those shapes disappear. Screening the vesicles in the chamber before and after temperature cycling results in a distinct difference in size distribution. Figure 3a,b show a schematic illustration of the convection chamber and the parabolic flow profile.

The convection driven flow chamber is constructed following the standard procedure shown in the methods section. Vesicles in the chamber travel in the double parabolic flow profile and experience position-dependent velocity and shear-forces. Figure 3c shows the simulated path of two vesicles in the convective flow. The absolute temperature difference was chosen smaller for the second case with elevated temperatures, as the viscosity of water is lower and, therefore, flow speed increases. In d) we show the vesicle volume distribution. When vesicles do not cross the phase transition temperature of their membrane lipids, the size distribution is shifted towards slightly larger volumes, suggesting smaller vesicles loosely adhering to larger ones. In the second case, vesicles shuttle through the chamber and across the phase transition temperature multiple times. Here, the size distribution is shifted to smaller vesicles, suggesting vesicle fission (see Fig. 3d).

In literature, there are several cases of vesicle fission described: vesicles can divide when shear forces are applied^13^ or when they are heated in a stationary case^12^. In our case we do only see the shift in size distribution towards smaller vesicle sizes when the transition temperature is exceeded. Combining these reported findings and the statistically significant shift to smaller vesicle sizes suggest the hypothesis of vesicle fission in the convection chamber. While this setup allows to study several thousand vesicles at the same time, tracking of single vesicles is difficult. To check whether indeed vesicle fission happens, we build a capillary setup mimicking the form of the flow profile and the temperature gradient of the convection chamber, however in a linear one-way version. Combined with lower vesicle density and higher magnification we study single vesicles on their way through the temperature profile.

**Figure 4:**
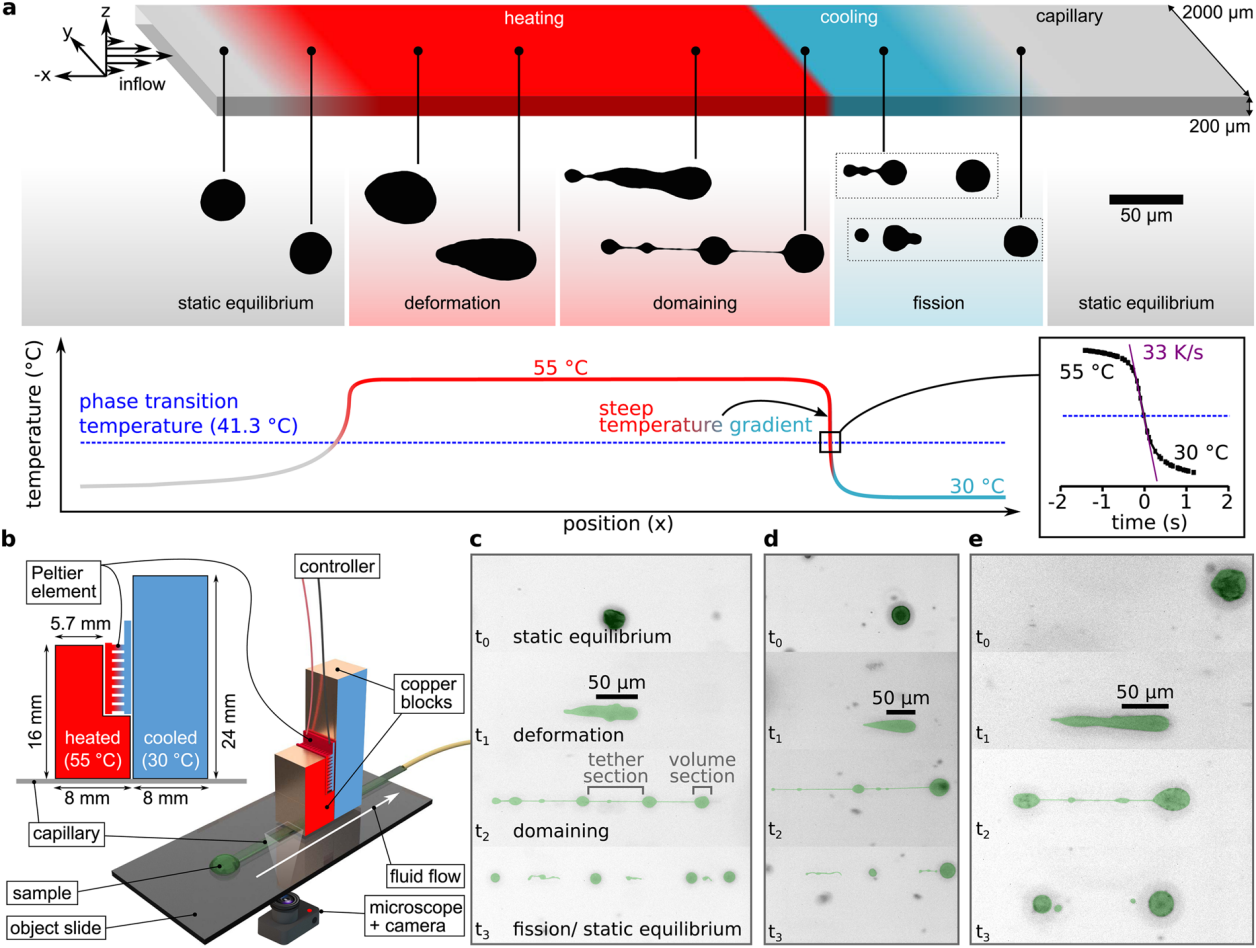
Capillary flow chamber to closely monitor vesicles during phase transitions. (**a**) Processed micrographs show four states of vesicle fission: at temperatures below the phase-transition temperature the vesicles are in static equilibrium and drift with the surrounding medium in the channel without being deformed by the shear force. In the second state, deformation, the vesicles are heated over their phase-transition temperature. The now deformable membrane gains additional surface area and transforms into rain-drop/ comet-tail like shapes. In state three, domaining, the combination of shear force and bending energy minimization leads to the formation of new stable shapes with volume and tether domains. In the phase fission the vesicle is divided by the membrane phase transition back to the gel-like state. The resulting vesicles are again in a spherical shape. The graph shows the temperature profile in the capillary as a function of the channel position. The zoom-in on the right shows the temperature experienced by a vesicle moving in the center stream line as a function of time with flow velocity = 550 µm/s (mean flow velocity: 367 µm/s). Maximum temperature gradients of up to 33 K/s are possible. This data is extracted from a COMSOL simulation. (**b**) Illustration of the setup: the copper blocks (red, blue) act as heat buffers and contact the capillary from the top. The Peltier element heats the L-shaped copper block and cools the other one. (**c**–**e**) Micrographs show vesicles that behave similar to the exemplarily chosen vesicle reshaping and fission in (**a**).

The experiments indeed show vesicle fission as seen in Fig. 4. Figure 4b shows the experimental setup with two copper blocks and a Peltier element for heating and cooling.

Figure 4a shows four states of vesicle deformation, that are reproduced in every fission event we monitored (N_fission_ = 55 out of N_total_ = 97). The first state is the *static equilibrium*. Here, the membrane is in the gel-like phase as the temperature is below the phase transition temperature. The surface area is in equilibrium with the enclosed volume – an outer force acting on the vesicle would lead to a strain in the membrane acting against that deformation. In the second state *deformatio*n the vesicle is in the hot part of the capillary and the temperature exceeds the main phase transition temperature of the lipid-membrane. As a result, the membrane expands suddenly while the vesicle volume remains approximatively constant. Additionally, the deformability increases as the bending modulus of the membrane decreases by a factor of about ten^23,24^. The exterior force caused by the parabolic fluid flow profile is now able to deform the vesicle. Due to the rectangular shape of the capillary the deformation in the z-direction is several times stronger than in the y-direction. The formerly rotationally symmetric (with regard to the x-axis) vesicle is not symmetric in this step and rather flat in the z-direction compared to the y-direction and elongated especially in the x-direction. In the third state *domaining* new lowest energy states of the vesicle shapes develop: volume containing sections are separated by tether sections of negligible volume. Volume containing sections are typically close to a spherical shape and basically encapsulate the entire vesicle volume (see Fig. 4c–e) with parts of the membrane area. The tube-shaped tether sections accommodate the remaining surface area and connect two neighboring volume sections. These vesicle shapes are stable under given conditions as no disruption has been observed and all vesicles adopt to a comparable shape in all performed experiments (for example see SI-Video).

In the fourth state *fission* the rapid cooling of medium and vesicle membrane below the phase transition temperature of the lipid membrane induces the phase transition from the fluid unordered phase to the gel-like membrane phase. The bilayer surface area shrinks back to its pre-phase transition state. At this state for slow temperature changes and low shear flow velocities the vesicle reshapes back to its spherical starting configuration. However, when a distinct threshold of shear flow velocity and temporal temperature gradient is reached, the tethers break apart. Breaking tethers open energetically unfavorable pores in the membranes of the volume domains. The vesicles close these pores by releasing inner volume into the surrounding medium, as *e.g*. shown earlier for endocytosis-like nanoparticle uptake^25,26^. By such shrinking to a smaller size and thus decreasing the surface-to-volume ratio, these new smaller vesicles are in a mechanical equilibrium state again. The resulting split-up vesicles are about equal in size in all our experiments (for example Fig. 4).

This mechanism represents a reproducible, controlled way to divide lipid vesicles with a structural membrane phase transition in microfluidic pore setups. Moreover, no processes other than a non-uniformal flow profile and a local temperature gradient are necessary for the division process. Most of the vesicle volume is preserved and still enclosed in the unilamellar bilayer vesicle. In control experiments under the same conditions DPPC was replaced by 1,2-dioleoyl-sn-glycero-3-phosphocholine (DOPC), a lipid lacking a phase transition in the respective temperature range. Here, not a single fission event was observed despite high deformability of the fluid membrane. Following Mercier *et al*.^16^ the unbalanced surface-to-volume ratio is key to vesicle division. The main phase transition of DPPC and the resulting increase in surface area provides this imbalance, while DOPC is lacking this property, explaining the lack of fission events described above. For deformed DPPC vesicles the fast phase transition back from fluid to gel-like state enables the vesicle division.

Furthermore, we assume the vesicles in the fluid phase to be equilibrated to the environmental conditions (temperature and shear force), when the front section of a tethered vesicle enters the steep temperature gradient as shown in Fig. 4. The immediate phase transition of the membrane with its accompanied decrease in area leads to a drastic increase in membrane tension. In turn, the increased tension leads to Marangoni flow of lipids from the tether and the rear vesicle section towards the front vesicle section^27^. This is accompanied by a flow of surrounding water, being dragged by the flowing lipid to the front vesicle section. This hinders volume flow towards the back vesicle section what becomes less important with increasing tether radius^27^.

Detailing the fission mechanism, we first investigate the energy necessary for a fission event.

### Energy required for fission

As the fission event requires the formation of two pores for the rupture of the tether (one in the volume section and one on the tether), we estimate an upper limit for the energy *E*_pore_ necessary for pore formation. We assume a pore radius of *r* = 25 nm and membrane tension $\sigma \approx 0$. Following Taupin *et al*.^28,29^

1 $${E}_{{\rm{pore}}}(r)=\gamma \cdot 2\pi \cdot r-\sigma \cdot \pi \cdot {r}^{2}$$

with the line tension $\gamma \approx 0.7\cdot {10}^{-11}\,{\rm{N}}\,$. This results in ${E}_{{\rm{pore}}}\approx {10}^{-18}\,{\rm{J}}$. However, for some finite membrane tension of about 1 mPa, *E*_pore_ can take a maximal value of ${E}_{{\rm{pore}}}\approx {10}^{-19}\,{\rm{J}}$ for r = 7 nm and becomes even negative for pore radii larger than *r* = 14 nm.

#### Energy supplied by a phase transition of the front vesicle

We can estimate the energy contributions of this transition following the approach of Heimburg^11^ as follows:

The contribution to the Gibbs Free Energy density due to area changes ${g}_{{\rm{A}}}$ is

2 $${g}_{{\rm{A}}}=\frac{1}{2}{K}_{{\rm{A}}}{(\frac{\varDelta A}{{A}_{0}})}^{2}$$

where ${K}_{{\rm{A}}}$ is the area compression modulus and $\Delta A$ the deviation from the equilibrium (here the gel-like phase) area ${A}_{0}$. Moreover,

3 $${K}_{{\rm{A}}}=\frac{1}{{\kappa }_{{\rm{A}}}^{T}}$$

with the area compressibility $\,{\kappa }_{{\rm{A}}}^{T}$.

**Figure 5:**
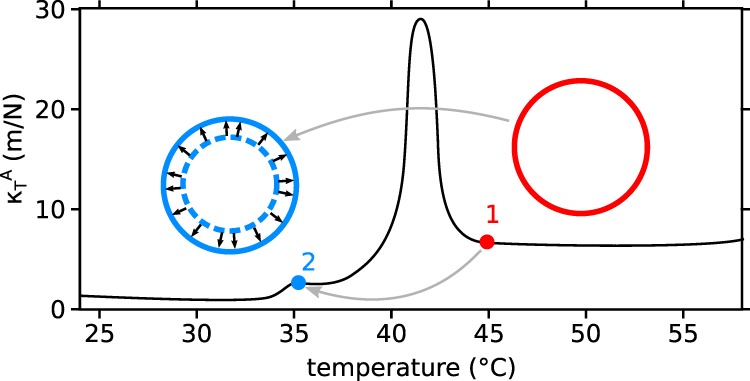
Model for energy estimation: area compression modulus as function of temperature. Prior to the temperature gradient the vesicle is at point 1, then quickly passes the transition temperature towards a lower temperature below the main transition (point 2). The vesicle keeps its geometry (blue solid circle) but is in the gel phase at around 35 °C with a smaller equilibrium size (dashed blue circle). The arrows indicate the membrane expansion resulting in membrane tension.

In our experiment prior to the steep temperature gradient from hot to cold the vesicle is in an equilibrium shape as a result of temperature and shear forces. For the following energy estimation, we illustrate the front vesicle of our dumbbell shaped vesicle as a spherical vesicle in Fig. 5.

**Figure 6:**
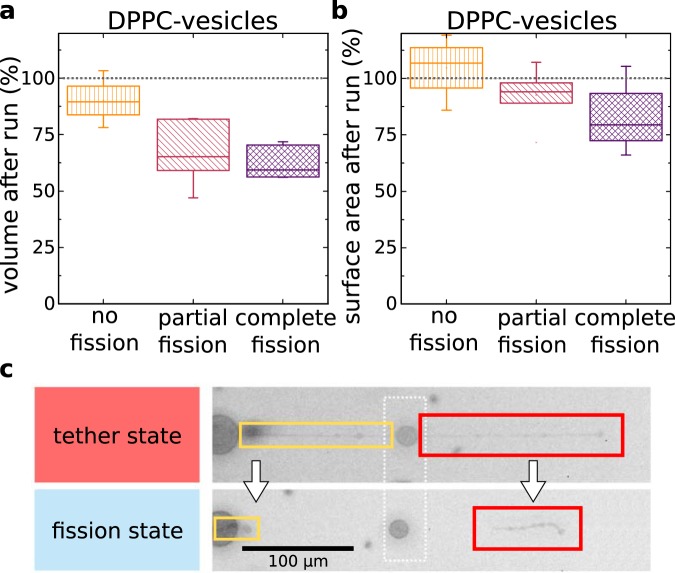
Vesicle volume and surface after fission and vesicles with broad phase transition, (**a**) Volume loss as a function of fission state: completely divided vesicles lose the most volume. Vesicles that do not fission do hardly lose volume. (**b**) Vesicle membrane area as function of fission state: vesicle lose less than a quarter of surface area by division. The loss of volume is geometrically necessary for the vesicle fission. (**c**) Microscope image of split off lipid tethers after vesicle fission. Therefore, surface area is lost. The yellow rectangle marks a tether still sticking to a spherical vesicle, the red rectangle marks a completely separated tether-section.

Starting from point 1 in the graph of area compression modulus as function of temperature, the vesicle quickly passes the transition temperature towards a lower temperature below the main transition (point 2). The vesicle keeps its geometry due to the incompressibility of water and only a minor chance to release volume from the interior during the short moment when the vesicle reaches the main transition temperature. Thus, at point 2 the vesicle still has the area of a fluid phase vesicle but is in the gel phase at around 35 °C. This is equivalent to a stretching of a gel phase vesicle (dashed line) to the size of a fluid phase vesicle (solid line). Thus, accepting the approximate volume conservation (see also Fig. 6), the energy difference between the points 1 and 2 is equal to a membrane expansion of about 25% using the ${{\rm{K}}}_{{\rm{A}}}(\,$35 °C).

Using Eqs. 2 and 3 with $\frac{\Delta A}{{A}_{0}}=\frac{{A}_{{\rm{fluid}}}-{A}_{{\rm{gel}}}}{{A}_{{\rm{gel}}}}\approx 25 \%$^11^, ${\kappa }_{{\rm{A}}}^{T}(35\,^\circ {\rm{C}})\approx 3\frac{{\rm{m}}}{{\rm{N}}}$ and ${A}_{{\rm{fluid}}}\approx 0.63\,{{\rm{nm}}}^{2}$ it follows:

4 $${g}_{{\rm{A}}}=\frac{1}{2}{K}_{{\rm{A}}}{(\frac{\Delta A}{{A}_{0}})}^{2}=\frac{1}{2}\cdot \frac{{\rm{N}}}{3\,{\rm{m}}}\cdot {0.25}^{2}=10\frac{{\rm{mN}}}{{\rm{m}}}=10\,\frac{{\rm{mJ}}}{{{\rm{m}}}^{2}}$$

5 $$\Delta {G}_{{\rm{A}}{\rm{\_}}{\rm{m}}{\rm{o}}{\rm{l}}}={g}_{{\rm{A}}}\cdot {A}_{{\rm{f}}{\rm{l}}{\rm{u}}{\rm{i}}{\rm{d}}}\approx \frac{3}{2}{k}_{{\rm{B}}}T$$

Where, $\Delta {G}_{{\rm{A}}\_{\rm{mol}}}$ and ${A}_{{\rm{f}}{\rm{l}}{\rm{u}}{\rm{i}}{\rm{d}}}\,$are the change in Gibbs Free Energy and area per molecule respectively. This energy can be compared with the energy necessary for the formation of a pore with radius *r*.

Thus, the Gibbs Free Energy $$\Delta {G}_{{\rm{A}}\_{\rm{ves}}}$$ for a typical vesicle with radius $$R=10$$ µm is $$\Delta {G}_{{\rm{A}}{\rm{\_}}{\rm{v}}{\rm{e}}{\rm{s}}}\approx 4\pi \cdot {(10{\rm{\mu }}{\rm{m}})}^{2}\cdot $$$$\frac{10\,{\rm{m}}{\rm{J}}}{{{\rm{m}}}^{2}}=1.2\cdot {10}^{-11}\,{\rm{J}} >  > $$ “*E*_pore_”. This energy easily suffices for arbitrarily large pores, even for vesicles that do not experience shear stress with membrane tension $$\sigma =0.$$

This estimation shows that all vesicles in our experiments could fission, if the pore formation energy would be the single decisive parameter. However, only a subset of vesicles experiences a fission in the capillary setup. Therefore, we investigate the influence of geometrical parameters like tether length, radius and flow velocity on the fission behavior.

First, we estimate the shear stress $\,\sigma$ using a mean shear rate for each vesicle depending on its particular position within the parabolic flow profile in the channel. In the experiments presented here the shear stress typically is ${\sigma }_{{\rm{shear}}}=\dot{\gamma \eta }\approx \frac{1}{{\rm{s}}}\cdot 1$ mPa s = 1 mPa. Fig. 6c shows boxplots of the estimated shear force *f* as a product of $\sigma$ and the total area of each vesicle for all vesicles categorized as *fission* and *no fission*. The median overall shear force $f$ for vesicles that do fission during the experiment and those that reshape without fission are ${f}_{{\rm{fission}}}=3.3$ pN and ${f}_{{\rm{no}}\_{\rm{fission}}}=1.8$ pN, respectively. Vesicles that fission do on average experience an 1.8-fold higher shear stress.

Second, we measure the mean tether radius from the fluorescence micrographs. Figure 6d shows boxplots of the tether radii for all vesicles categorized as *fission* and *no fission*. The median apparent tether radii $\rho$ for vesicles that are divided during the experiment and those that reshape without fission are ${\rho }_{{\rm{fission}}}=1.1$ µm and ${\rho }_{{\rm{no}}\_{\rm{fission}}}=1.3$ µm, respectively. This is in line with the argumentation on Marangoni flow as discussed above, especially concerning the dependence of flow resistance from the radius $\sim {\rho }^{4}$. The mean difference of 15 % in radius results in a flow resistance difference by a factor of two.

**Figure 7:**
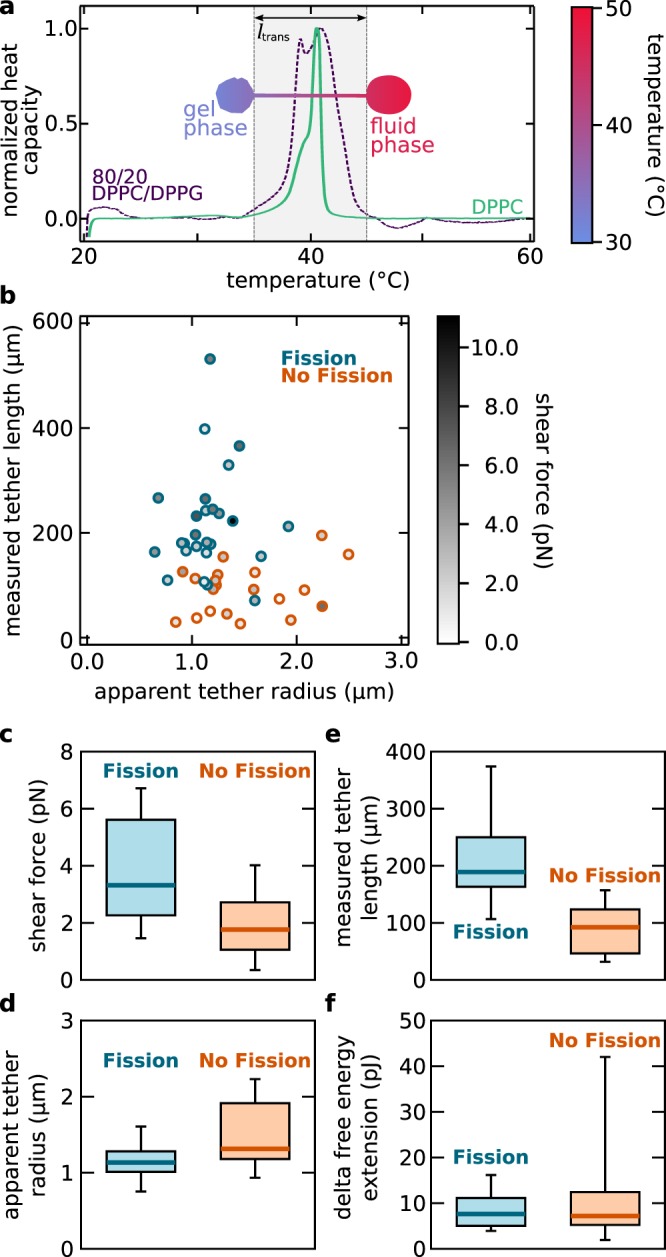
Tether length and tether radius are the important parameters determining fission. (**a**) Vesicles made from DPPC and DPPG (80:20 ratio) experience a broader phase transition than vesicles made form DPPC only. The vesicle drawn as an overlay (blue-red color map) shows the temperature distribution over a vesicle moving with a velocity of 550 µm/s. (**b**) Measured tether length as a function of apparent tether radius. Vesicles that fission are marked blue, vesicles that do not fission are marked orange. Fission occurs more often for large tether lengths and small tether diameters. The grey scale filling of the symbols indicate the total shear force experienced by each vesicle. (**c**–**f**) Boxplots visualize the distribution of shear force, tether length, tether radius and $\Delta {G}_{{\rm{A}}{\rm{\_}}{\rm{v}}{\rm{e}}{\rm{s}}}$ categorized as fission and no fission. Boxes include 50 % of data points, the thick horizontal line marks the median and whiskers mark the range of 80 % of the data range.

Third, we measured the tether lengths from the fluorescence micrographs immediately before the vesicles pass the steep temperature gradient from the hot to the cold region. Figure 7e shows boxplots of the tether length for all vesicles categorized as *fission* and *no fission*. The median tether length $l$ for vesicles are ${l}_{{\rm{fission}}}=189$ µm and ${l}_{{\rm{no}}\_{\rm{fission}}}=92$ µm. Therefore, we see a strong correlation of tether length and fission events. Long tethers are prone to fission.

Defining the mean value of ${l}_{{\rm{fission}}}$ and ${l}_{{\rm{no}}\_{\rm{fission}}}$ as the transition tether length ${l}_{{\rm{trans}}}$ we can compare ${l}_{{\rm{trans}}}$ with the width of the phase transition: the setup provides a steep temperature gradient of 0.07 K/µm at the phase transition region. Thus, the transition tether length ${l}_{{\rm{trans}}}$ corresponds to a temperature difference of $\Delta T=10$ K. This $\Delta T$ is about 2.3 times the FWHM of the phase transition temperature width of the used lipid mixture, see Fig. 7a. Even more convincing, 10 K is almost exactly the width of the complete phase transition width.

Summing this discussion up, we conclude that shear forces acting on the vesicles result in a shape change with the formation of a tether of distinct length and radius. The tether radius and length determine the relaxation dynamics of membrane tension after the phase transition of the front vesicle section. In combination with the temperature gradient in the capillary these parameters then determine the Gibbs Free Energy change, that finally leads to the fission event. Or in even simpler words: the shear force decides about the fission indirectly while the phase transition delivers the energy for the fission.

As DOPC-membranes do not have a phase transition in this temperature range, DOPC-vesicles do not change their shape irreversibly and no fission event can be detected. This also holds for vesicles from cholesterol-DPPC mixtures (≥30% cholesterol, see SI). Thus, the capillary setup experiments proof our hypothesis concluded from the shift in size distribution in the convection driven chamber: a robust vesicles fission takes place under the influence of shear flow and a temperature gradient reaching across a phase transition temperature of the membrane.

Important for protocells, especially for early earth scenarios, is the conservation of its building blocks. In a dilute ocean shell material as well as encapsulated material were scarce^1,2,30^. In Fig. 6 the mean volume and surface of the vesicles before and after passing the capillary are shown. Due to the setup geometry only the 2D-projections in the x-y-plane are analyzed. Vesicles are assumed to be rotationally symmetric. Measurements of vesicles in the capillary setup are classified in three categories: no fission, partial fission and complete fission. Here, partial fission summarizes all irreversible shape deformations including incomplete separation of tether and daughter vesicles.

The total vesicle volume decreases only slightly when there is no fission, decreases for about 35% at partial fission and for about 40% for complete fission. An ideal fission event would not lose any surface area. But the resulting daughter spheres of course have a combined volume smaller than the initial volume. For two and three equally sized daughter vesicles this decrease of volume is about 30% and 42%, respectively. Thus, the fissioned vesicles retain roughly as much volume as geometrically possible (analytical estimation shown in SI).

Along the same line, surface area remains constant if the vesicles do not fission. The slight increase in Fig. 6b points towards a systematic error in the calculation of surface and volume from the 2D-projection (e.g. selection error in fluorescent micrograph). For partial and complete fission, the surface area decreases up to 10% and 25%, respectively. Partially this is due to split off tether-sections as shown in Fig. 6c.

## Conclusion

The mechanism described here shows a division pathway that combines a steep temperature-gradient and a parabolic flow profile, that reliably divides lipid vesicles. Without the need for additional, chemically induced processes, vesicles with a first order membrane phase transition are prone to fission by this mechanism. In a thermal convection chamber we find a significant reduction of the size distribution for a wide range of vesicle sizes. Tracing single vesicles in a capillary we monitored vesicle shape transformations resulting in fission. From these observations we deduce the underlying mechanism: domaining due to the experienced shear forces causes a separation of vesicle volume by tether-like surface domains which rupture during the phase transition back to the gel-like phase.

It would be highly interesting to extend existing theoretical models from Canham, Helfrich, Evans, Seifert, Miao and Döbereiner^31–38^ to vesicles experiencing force fields in a scenario as shown here including the non-linear behavior due to membrane phase transitions. Developing this idea further should also consider the sensitivity of these phase transitions to interaction with the enclosed material^39^ like proteins or RNA. The temperature cycling is one of the two driving forces of this simple proto-cell division mechanism. But the combination of DNA in the proto-cell with these temperature oscillations can even melt double stranded DNA into single strands and make them accessible for replication mechanism as proposed by Mansy and Szostak^40^. In combination with the fission mechanism discussed in this paper a full cell cycle with included information distribution might be provided.

In a context of Origin of Life, vesicles could be a step on the way to proto-cells that enclose, replicate, transport, shield, and distribute information, *e.g*. in the form of nucleic acids. Without the sophisticated division mechanisms of evolved modern cells, a much simpler and reliable mechanism for proto-cell division is necessary to have a complete cell cycle of formation, information-inclusion, division, and growth for lipid vesicles. Also, both capillary and convection chamber are experimental realizations of hydrothermal microenvironments. They combine non-uniform flow profiles with rapid temperature-changes, a setting which is plausible in the context of the Origin of Life and is known to have the ability to accumulate dissolved molecules from strongly diluted solutions and provide a mechanism for the formation of lipid-vesicles, a structure that is widely seen as a proto-cell candidate.

Prebiotically plausible protocells probably had multiple different membrane molecules. Membrane phase transitions would not have been as sharp as shown here. The environment in our experiments is also very controlled. Loosening those parameters would probably lead to a lower fission efficiency or the emergence of new division mechanisms which might show similar characteristics. However, the same physics as for our protocell model with a structurally simple membrane would apply. Taken together, a robust fission mechanism can arise from simple gradients, fluid flow and non-linear mechanical membrane properties, as present at temperature induced membrane phase transitions.

## Methods

### Vesicle preparation

In this paper, we use Giant Unilamellar lipid Vesicles (GUV) with a diameter of up to 100 µm. Their size is comparable to eukaryotic cells^41^. The vesicle formation in the laboratory is achieved by the electro-swelling method^42^: 15 µl of 10 mM dipalmitoylphosphatidylcholine (DPPC) dissolved in chloroform is spread on indium tin oxid (ITO) coated glass. The drop is spread out in between the conductive surfaces of the ITO coated glass plates by pressing them together. They are separated by pulling them away from each other in the plane of slide. The organic solvent is evaporated in a vacuum for 6 h. Two glass slides and a teflon spacer form an electro-swelling chamber. The conductive ITO surface is connected to a frequency generator to induce an oscillating electric field of 1 mV/mm with a frequency of 10 Hz for 6 hours between the two glass slides. Afterwards, the swelling chamber is placed in a water bath with a temperature above the lipids phase transition temperature (for DPPC: 52 °C).

### Tracking vesicles in the capillary setup

The vesicles were studied using a *Zeiss Axiovert 200* fluorescence microscope and a *Hamamatsu Orca* camera. The motorized stage of the microscope is controlled with a joystick-controller. The stage is moved at the same speed of the vesicle but in the opposite direction. This keeps the vesicle in the center of the field of view. The movie in the Supporting Information is additionally stabilized using the open-source *Blender*-software.

### Convection chamber and capillary setup

The bottom of the front opening of the capillary (*CM Scientific Ltd*., 200 µm by 2000 µm made of borosilicate glass) is glued (*Sekundenkleber Gel, UHU*) to an object slide. The copper contacts are fixed to the object slide with hot glue. A tube is fixed to the back of the capillary with a shrink-tube. A pressure difference reservoir is attached to the tube and enables to suck the sample through the capillary. The heating is done with a Peltier element controlled by a TEC control software from *Meerstetter Engineering*. The convection chamber is 3D printed with a visible light-curing resin on a self-made illumination station. The resin part is pressed in between a silicon wafer (bottom, cold side) and a sapphire crystal (top, hot side). The sapphire is optically transparent and allows to monitor the inside of the chamber, *e. g*. the vesicles. The sapphire is also a very good heat conductor with a heat conductivity up to 25 W/mK. The heating is done by two copper contacts and a resistance heater and controlled by a *LabView* program. The back side is cooled by a *Julabo CORIO CD-300F* liquid cooling bath.

### Simulation of fluid-flow stream lines

The paths of vesicles inside the convection chamber are simulated with a combination of *COMSOL* and Brownian motion simulation: *COMSOL* simulates the fluid flow inside the chamber due to the temperature gradient. This fluid flow map is overlaid by a Brownian motion simulation done in C and *LabVIEW*, as reported earlier^4^.

### Image transformation for size distribution

In contrast to the capillary setup where a single vesicle is followed with the motor stage and camera of the microscope, in the chamber setup the entire chamber is scanned. Vesicles are imaged without convective flow, before and after thermo-induced convective cycling. A simple threshold filter creates a binary version of the images with white (0) background and black (1) vesicles. The visualization by accumulated integration has the advantage of not being influenced by a bin size selection as it is the case in standard histograms. The normalization allows to compare the size-distribution of two scans of the same sample before and after temperature cycling.

## Supplementary information

Supplementary Material

Supplementary Video
