# Supplementary material for: Fission of Lipid-Vesicles by Membrane Phase Transitions in Thermal Convection
Source: Sci Rep. 2019 Dec 11;9:18808. doi: 10.1038/s41598-019-55110-0 (PMC6906453; doi:10.1038/s41598-019-55110-0)
Supplement: Supplementary file 1 — Supplementary Material [file 41598_2019_55110_MOESM1_ESM.docx]

Fission of Lipid-Vesicles by Membrane Phase Transitions in Thermal Convection

Patrick W. Kudella^1,2,3^, Katharina Preißinger^1^, Matthias Morasch^2,3^, Christina F. Dirscherl^2,3^, Dieter Braun^2,3^, Achim Wixforth^1,2^, Christoph Westerhausen^1,2,4*^

^1^Chair for Experimental Physics I, University of Augsburg, Germany

^2^Center for NanoScience (CeNS), Ludwig-Maximilians-Universität, Munich, Germany

^3^ Systems Biophysics, Ludwig-Maximilian University Munich, Munich, Germany

^4^Chair for Physiology, University of Augsburg, Augsburg, Germany

^*^ christoph.westerhausen@gmail.com

Supplementary Information - Table of Contents

[Flow profile in a capillary 2](#_Toc22731028)

[Thermally driven convection trap 3](#_Toc22731029)

[Surface- and Volume-Change 4](#_Toc22731030)

[Dependency of fission on the vesicle size 4](#_Toc22731031)

[Vesicles without phase transition do not fission 5](#_Toc22731032)

[DOPC vesicles with equilibrated membrane to surface area 5](#_Toc22731033)

[DPPC + cholesterol and DOPC with excess membrane area 6](#_Toc22731034)

[SI-Literature 8](#_Toc22731035)

# Flow profile in a capillary

A narrow glass capillary (*VitroCom VitroTubes™*) with an inner width of 2000 µm and an inner height of 200 µm is used as flow channel resembling e.g. a crack in volcanic rock. Porous rocks (thermal vents) at the bottom of the sea are common on prebiotic earth and provide strong temperature gradients with lava-heated stone on the one side and cool ocean water at the other.


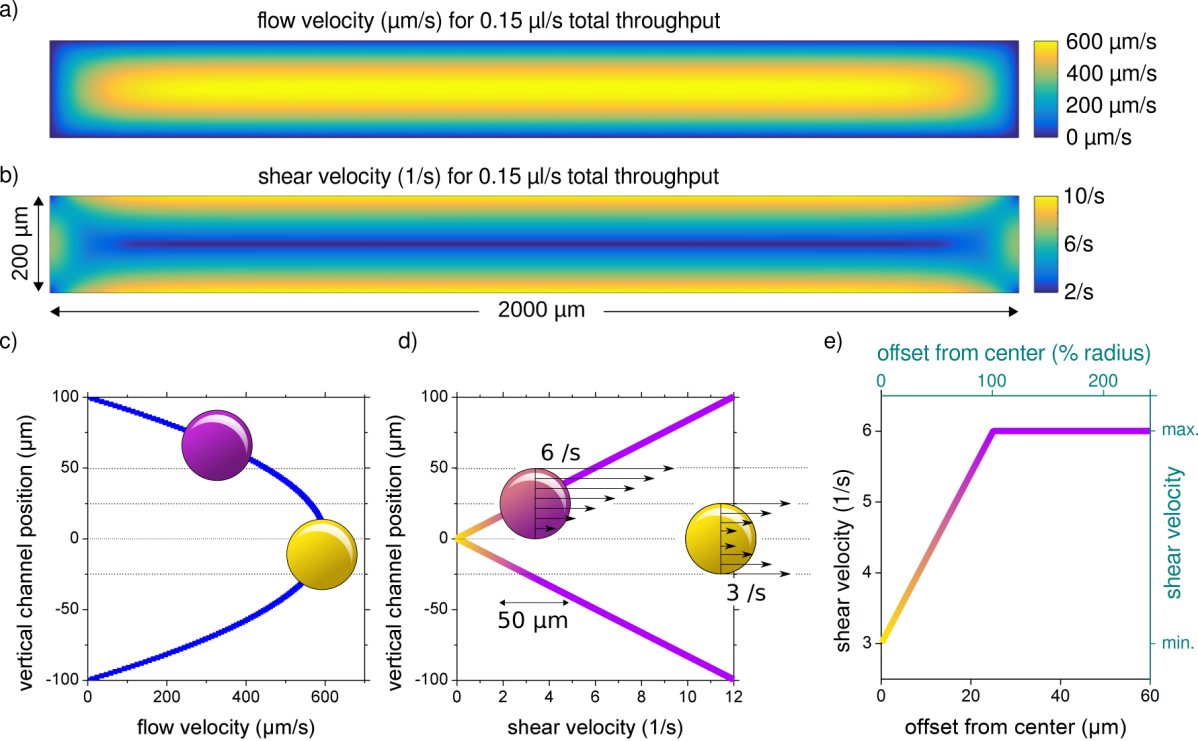


**SI-Figure 1,**

**a)** Cross section of the rectangular capillary used in the experimental setup: Flow velocity.

**b)** Shear velocity.

**c)** Vertical flow profile in the capillary, vesicles with diameter of 50 µm as reference size.

**d)** Vertical shear velocity profile in the capillary as a function of the channel position. Again, vesicles with a diamater of 50 µm ar shown as a reference. The black arrows show the shear velocity depending on the vesicle position in the channel. Due to the rectangular shape of the capillary the flow velocity is limited by its low height.

**e)** Shear velocity for 50 µm diameter vesicle as function of vertical offset from the center of the channel.

In SI-figure 1 a cross-section of the rectangular capillary is shown. For a total throughput of 0.15 µl/s flow velocities of up to 600 µm/s are reached. The flow velocities are calculated for the given shape of the capillary with a Matlab^©^-script^1,2^. The vertical flow profile shows the parabolic velocity distribution as a function of vertical channel position. The flow velocity is restricted by the smallest dimension of the capillary cross-section (z-direction) caused by the parabolic flow profile. Due to the velocity change as a function of position inside the channel, bigger objects experience a stronger shear force in the flow. This force is given by the shear velocity times the viscosity of the fluid. Vesicles that are located completely in the upper or lower half of the channel, experience the maximum possible shear force in the setup due to the symmetry of the flow profile.

For a vesicle radius of 26 µm, a mean fluid velocity of 400 µm/s at 35 °C, and a dynamic viscosity of 0.7191 mPas^3^ the shear force can be as high as 4.31 mPa.

As a test-assembly the channel is glued on a glass object slide. The temperature in the channel is regulated using a Peltier element. The Peltier element is fitted between two copper blocks which act as heat buffers and contact the capillary on the topside.

# Thermally driven convection trap

In thermally driven convection traps vesicles can be formed by self assembly. In the prebiotic ocean the local concentration of dissolved molecules like lipids was very low but could be increased by multiple folds with the interplay of convection and the thermophoretic effect in porous volcanic rock. An artificial convection chamber with a strong temperature gradient is used as a second, statistical setup for the vesicle fission analysis. In contrast to the capillary setup where only one vesicle is monitored at a time the convection setup allows to monitor a greater number of vesicles.


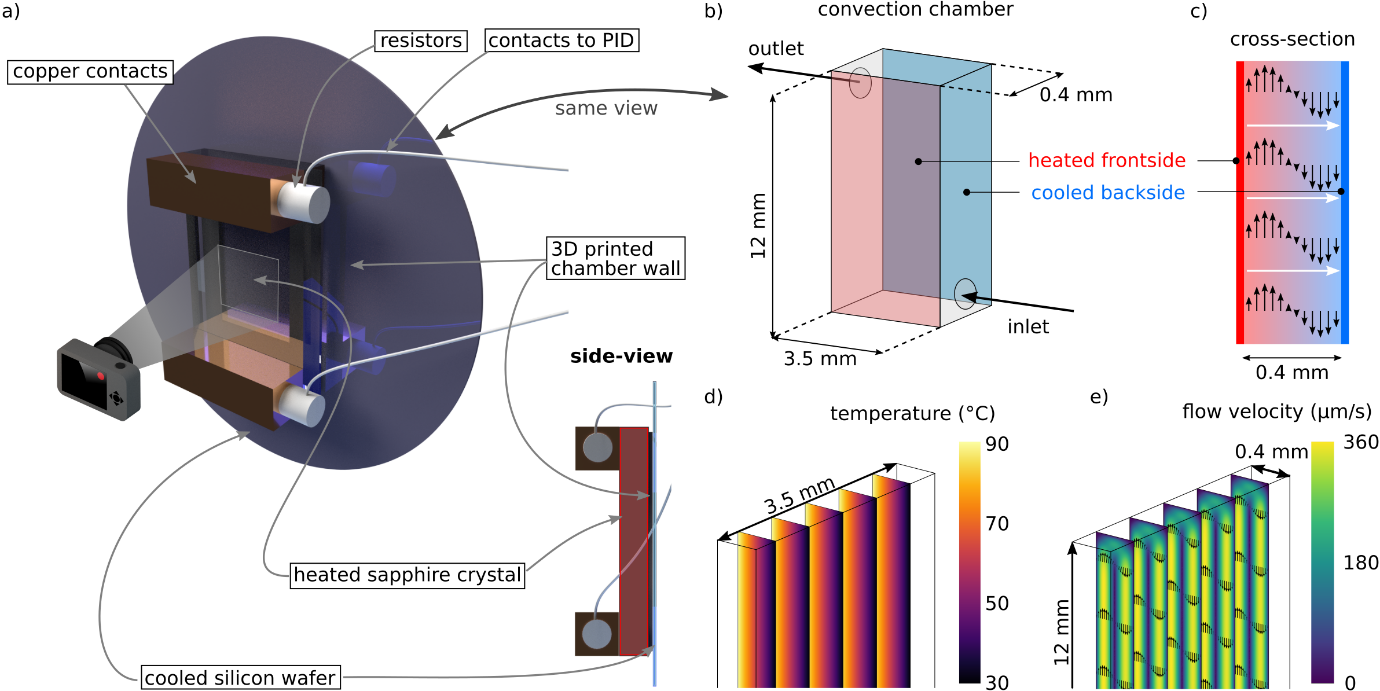


**SI-Figure 2**,

**a)** Render of the chamber assembly with a cooled wafer on the backside and two heated copper contacts. The sapphire crystal is transparent and a very good heat conductor. The chamber walls are 3D-printed and sandwiched between the sapphire and the silicon wafer.

**b**) Schematic drawing of the convection chamber with heated front side and cooled backside.

**c)** The chamber cross-section shows the parabolic flow profile (black arrows) and the path of thermophoresis (white arrows) from hot to cold side.

**d)** Numerical simulation of the temperature distribution in the convection chamber employing COMSOL^©^ FEM-simulation.

**e)** Simulated flow velocity in the chamber. The values aquired by FEM simulation are similar to the capillary setup shown above.

As shown in SI-figure 2 b) the convection chamber is 3.5 mm wide and 12 mm high. The depth of 400 µm is two times the height of the capillary in the setup for single vesicle monitoring as in figure 4 of the preliminary experiment stated above. A temperature gradient is applied by a heated sapphire at the front and a cooled silicon wafer at the backside (the sapphire is contacted with two copper blocks that house heating resistors, the wafer is cooled with a *Julabo CORIO CD-300F* refrigerated circulator).

In SI-figure 2 d) the temperature field for an applied gradient of 90 °C to 30 °C is simulated with COMSOL^©^. The resulting flow velocity is similar to the flow profile of the capillary setup shown above. The temperature gradient along the flow field depends on the vesicle position in the chamber with up to 30 K/s compared to 33 K/s in the capillary setup.

In the experiment, vesicles are first loaded into the chamber using a pressure pump. With a *Zeiss Vario* microscope, *Allied Vision Stingray* camera and *Mitutoyo* long working distance 10x objective the chamber is scanned through the transparent sapphire crystal over its whole height. Then the temperature regulation for front and back is switched on for 10 minutes leading to circular convection in the chamber and a mean flow velocity of 240 µm/s at a gradient of 90 °C to 30 °C. Thus, the vesicles undergo the phase transition up to 11 times in 10 minutes, depending on their position in the flow profile. Afterwards, the chamber is scanned again. The size distribution of the enclosed vesicles is compared before and after the temperature induced vesicle convection.

# Surface- and Volume-Change

In a geometrical consideration, a sphere that gets divided and keeps its surface must loose volume, in case of two equally sized daughter vesicles. The mother vesicle has volume *V*_1_, surface area *A*_1_ and radius *r*_1_. The daughter vesicles have both the volume *V*_2_ = *V*_21_ + *V*_22_, surface area *A*_2_ = *A*_21_ + *A*_22_ and the same radius *r*_2_ = *r*_21_ = *r*_22_.


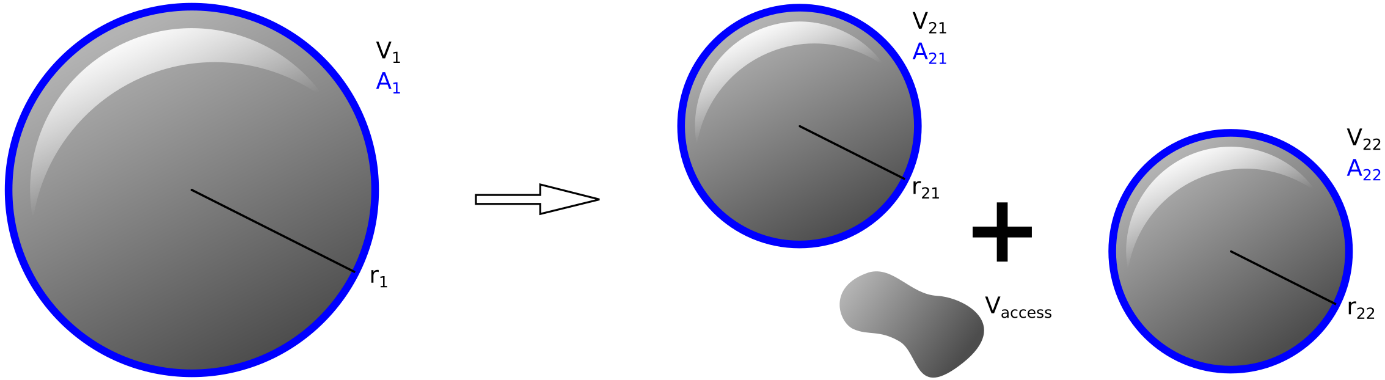


**SI-Figure 3**, a spherical vesicle is divided into two vesicles with the same surface area. The two smaller vesicles can not enclose the entire volume of the vesicle before division for geometrical reasons.

$$A_{1}=4\pi r_{1}^{2}\to V_{1}=\frac{4}{3}\pi r_{1}^{3}$$

$$A_{1}=A_{2}= A_{21}+A_{22}=4\pi r_{21}^{2}+4\pi r_{22}^{2}\to r_{21}^{2}+r_{22}^{2}=2r_{2}^{2}=r_{1}^{2}\to r_{2}=r_{1}*\sqrt{0.5}$$

$$V_{2}=V_{21}+V_{22}=\frac{4}{3}\pi\left( r_{21}^{3}+r_{22}^{3} \right)=\frac{4}{3}\pi\left( {2r}_{2}^{3} \right)=\frac{4}{3}\pi\left( 2*r_{1}^{3}*\sqrt{0.5}^{3} \right)\cong\frac{4}{3}\pi r_{1}^{3}*0.71$$

$$V_{2}=V_{21}+V_{22}\cong V_{1}*0.71\to V_{2}+V_{access}=V1 .$$

In figure 6 in the main manuscript the volume and surface of the vesicles are measured before and after they are flushed through the capillary setup. Due to the setup geometry only the 2D-projections are visible and are assumed to be rotationally symmetric as they are in the “static equilibrium” phase. Measurements of vesicles in the capillary setup are classified in three categories: no fission, partial fission and complete fission. Partial or no fission occurs when, for example, the flow velocity was to small in the experiment. If the vesicles do not fission, the surface area seems to slightly increase, which points towards a systematic error in the calculation of surface and volume from the 2D-projection. For partial and complete fission, the surface area decreases up to 25 % from the initial value due to completely split tether-sections as shown in Figure 6 c). Here, tether sections can remain explaining the loss of surface area. The volume follows the trend of the surface area and decreases only slightly when there is no fission, about 35 % at partial fission and about 40 % for complete fission.

## Dependency of fission on the vesicle size

The volume or surface area of the vesicles might be expected to have the strongest influence on the fission success. But analyzing the size as the only parameter, we find that it doesn’t show the correlation we assumed. Therefore, we took other parameter into account as shown in the results section in the paper. In SI-Figure 4 we show fission and no fission events as function of their size (here the surface area – we argue in the paper, that vesicles start in an equilibrium of surface to volume, therefore, area is a valid size estimation and comparison parameter).


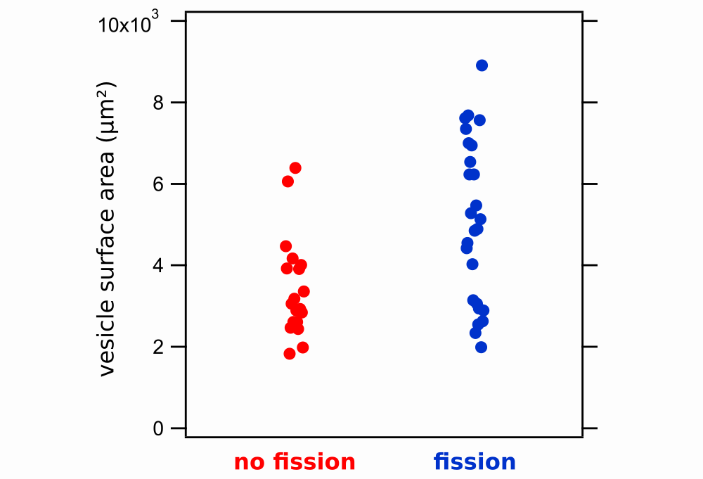


**SI-Figure 4, Vesicle fission as function of the vesicle size:**
although size being the most obvious paramter, the success of fission does not depent on the vesicle size alone. Therefore, there is no correlation of that paramter compared to fission and no fission events.

# Vesicles without phase transition do not fission

## DOPC vesicles with equilibrated membrane to surface area

The most important characteristics leading to lipid vesicle fission are the non-uniform flow profile and the phase transition. On the one hand, the fluid flow enables steep temperature gradients in the capillary: without artificial heating and cooling, as necessary in static experiments, this setup is comparable to volcanic rock in deep sea thermal vents. On the other hand, the non-uniform flow profile induces shear forces on the vesicles and deforms them in their fluid unordered membrane phase.

Here, the phase transition is crucial, since it allows for the vesicle's deformation and for the reshaping by the transition to gel-like, ordered membrane state. To test this criterion of phase transition, vesicles made from dioleoylphosphatidylcholine (DOPC) are used in the same capillary setup: Over the whole temperature range of the setup DOPC-vesicles are in the liquid-like unordered state and do not experience a phase transition. As expected, the vesicles are sphere-shaped without flow. In the flow profile of the capillary the vesicles are deformed but not divided at the temperature drop.


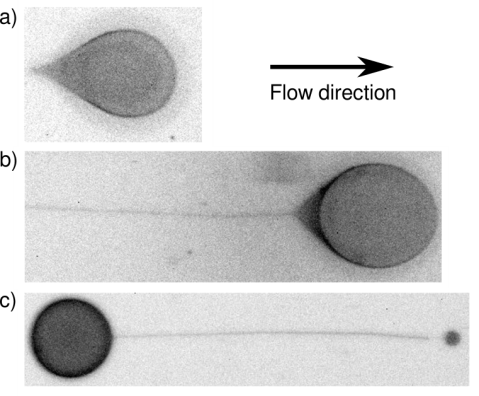


**SI-Figure 5, DOPC vesicle in capillary:**

**a)** Drop-like reshape of a DOPC vesicle in fluid flow.

**b)** Tethering of the vesicle membrane.

**c)** Tethering of a vesicle with two volume domains and one membrane domain.

In SI-figure 5 DOPC vesicles show deformation due to the fluid flow in the capillary. SI-figure 5 a) the vesicle forms a droplet but no significant reshaping like the DPPC vesicles in figure 4 in the main manuscript. In contrast to DPPC vesicles, DOPC vesicles do not feature an imbalance of surface and volume and therefore are stable. The gain of membrane surface of DPPC vesicles during their phase transition enables the domaining phase and thus the reshaping into volume and surface domains, which is not the case for DOPC vesicles. In no single experiment DOPC vesicles were divided while passing the sharp temperature gradient, as stated in the main manuscript.

In conclusion, the phase transition is essential for the reshaping of the vesicle in the fluid flow. The gain in membrane surface allows for reshaping and the loss of surface area during the second transition enables the complete division of lipid vesicles.

## DPPC + cholesterol and DOPC with excess membrane area

To investigate the importance of a phase transition for efficient and fast membrane area expansion and contraction, we prepared vesicles made from DPPC and cholesterol. These vesicles show nonlinear membrane area expansion at the temperature where DPPC has its main phase transition. The phase transition of these mixtures gets weaker with more cholesterol included in the membrane as shown in publications^4,5^. We also used DOPC vesicles with a suface to volume inbalance by osmotic pressure difference: 60 mM on the inside, about 400 mM sucrose in the outside. Those vesicles do not change their surface area as drastically as DPPC^6^ but allow for reshaping of the vesicle.

In SI-Figure 6 we show a selection of still images from the capillary. DPPC with 30 mol% and 40 mol% cholesterol show elongation into elipsoidal or teardrop-like shapes. They might also drag a tail made of the outer lipid membrane leaflet, as known from DOPC (see above). When the vesicles shuttle through the capillary, the reshaping happens in the heated regime (55 °C) as expected. In the cooled regime (30 °C) the vesicles tend to reverse completely into a spherical shape. We did not observe any fission.

DOPC vesicles with excess membrane area show similar outer leaflet tails. They also show complex reshaping as known from DPPC in the main manuscript from initially spherical to dumbell-like shapes with two volume sections, that are connected by a tether. Anyhow, as there is no mechanism for a decrease of membrane area as in DPPC, those vesicles remain in their new shape and do not fission.


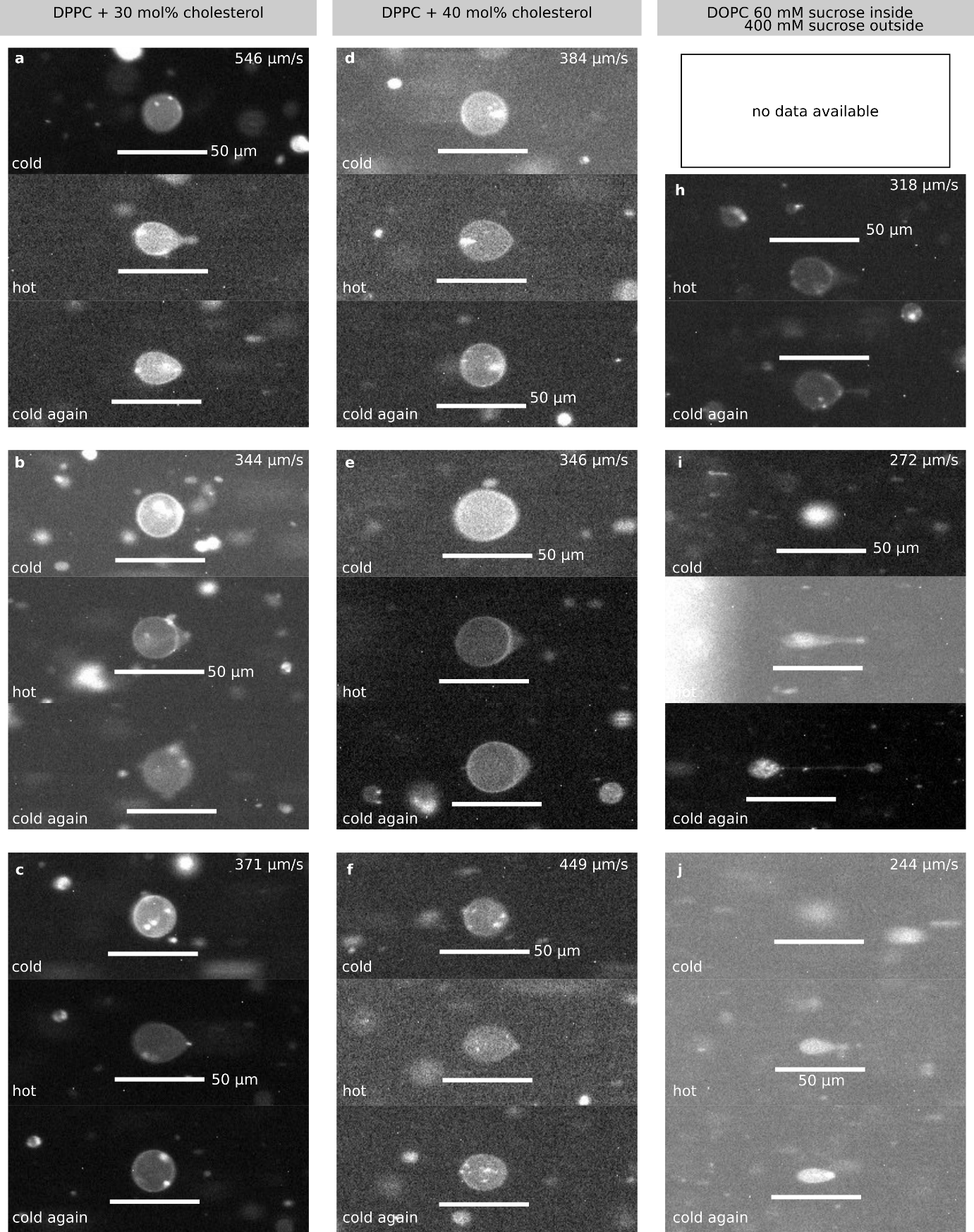


**SI-Figure 6,** **DPPC with cholesterol and DOPC with excess area:**

**column one:** vesicles with DPPC and 30 mol% of cholesterol. Vesicles show three kinds of reshaping, slight tethering with backforming (**a**), a tail made from excess outer leaflet membrane area (**b**) and elongation with backforming (**c**).
**column two:** vesicles with DPPC and 40 mol% of cholesterol. Vesicles show elongation with backforming (**d**, **f**) and a tail made from excess outer leaflet membrane area (**e**). here, we observed no tethering, probably due to the smaller membrane area expansion.
**column three:** vesicles made from DOPC with an inner sucrose concentration of 60 mM and an outer sucrose concentration of about 200 mM (**h**) and 400 mM (**i**, **j**). The osmotic pressure difference leads to a decreased vesicle volume but no decrease in membrane area. Therefore, the excess membrane area allows reshaping of the vesicle into shapes similar to DPPC only. In contrast, these vesicles here do not fission in the cold regime.

# SI-Literature

1. Breyer, D. From single to bundles - thermodynamic activation and adhesion of von Willebrand factor. (Dissertation to be completed, Universität Augsburg).

2. Tabeling, P. *Introduction to Microfluidics*. (Oxford University Press, 2005).

3. Release on the IAPWS Formulation 2008 for the Viscosity of Ordinary Water Substance. in *International Association for the Properties of Water and Steam* (2008).

4. Redondo-Morata, L., Giannotti, M. I. & Sanz, F. Influence of cholesterol on the phase transition of lipid bilayers: A temperature-controlled force spectroscopy study. *Langmuir* (2012). doi:10.1021/la302620t

5. Ipsen, J. H., Mouritsen, O. G. & Bloom, M. Relationships between lipid membrane area, hydrophobic thickness, and acyl-chain orientational order. The effects of cholesterol. *Biophys. J.* (1990). doi:10.1016/S0006-3495(90)82557-1

6. Aghaaminiha, M. & Sharma, S. Spatial Distribution of Cholesterol in Lipid Bilayers. *bioRxiv* 636845 (2019). doi:10.1101/636845
